# Urinary Epidermal Growth Factor as a Marker of Disease Progression in Children With Nephrotic Syndrome

**DOI:** 10.1016/j.ekir.2019.11.018

**Published:** 2019-12-05

**Authors:** Debbie S. Gipson, Howard Trachtman, Anne Waldo, Keisha L. Gibson, Sean Eddy, Katherine M. Dell, Tarak Srivastava, Kevin V. Lemley, Larry A. Greenbaum, Sangeeta Hingorani, Kevin E. Meyers, Frederick J. Kaskel, Kimberly J. Reidy, Christine B. Sethna, Cheryl L. Tran, Chia-shi Wang, Katherine R. Tuttle, Gia Oh, Alicia M. Neu, Elizabeth Brown, Jen-Jar Lin, Jennifer Lai Yee, Therese M. Roth, Jonathan P. Troost, Brenda W. Gillespie, Matthew G. Sampson, Matthias Kretzler, Wenjun Ju

**Affiliations:** 1 Division of Nephrology, Department of Pediatrics, University of Michigan, Ann Arbor, Michigan, USA; 2 Division of Nephrology, Department of Pediatrics, New York University Langone Health, New York, New York, USA; 3 University of North Carolina Kidney Center at Chapel Hill, Chapel Hill, North Carolina, USA; 4 Department of Pediatrics, Case Western Reserve University, Cleveland Clinic Children's, Cleveland, Ohio, USA; 5 Section of Nephrology, Children's Mercy Hospital and University of Missouri at Kansas City, Kansas City, Missouri, USA; 6 Division of Nephrology, Children's Hospital-LA, Los Angeles, California, USA; 7 Division of Pediatric Nephrology, Department of Pediatrics, Emory University and Children's Healthcare of Atlanta, Atlanta, Georgia, USA; 8 Department of Pediatrics, University of Washington School of Medicine, Seattle, Washington, USA; 9 Division of Nephrology, Seattle Children's Hospital, Seattle, Washington, USA; 10 Division of Nephrology, Department of Pediatrics, Children's Hospital of Philadelphia, Philadelphia, Pennsylvania, USA; 11 Division of Nephrology, Department of Pediatrics, Albert Einstein College of Medicine and Montefiore Medical Center, New York, New York, USA; 12 Pediatric Nephrology, Cohen Children's Medical Center of New York, Zucker School of Medicine, Hofstra University, Hempstead, New York, USA; 13 Department of Pediatrics, Mayo Clinic, Rochester, Minnesota, USA; 14 Providence St. Joseph Health, Providence Medical Research Center, Spokane, Washington, USA; 15 Department of Internal Medicine, University of Washington, Seattle, Washington, USA; 16 Division of Nephrology, Department of Pediatrics, Stanford University School of Medicine, Stanford, California, USA; 17 Division of Pediatric Nephrology, Department of Pediatrics, Johns Hopkins University School of Medicine, Baltimore, Maryland, USA; 18 Division of Nephrology, Department of Pediatrics, UT Southwestern Medical Center, Dallas, Texas, USA; 19 Division of Pediatric Nephrology, Brenner Children's Hospital, Wake Forest University Baptist Health, Winston-Salem, North Carolina, USA; 20 Division of Nephrology, Boston Children’s Hospital, Boston, Massachusetts, USA

**Keywords:** disease progression, epidermal growth factor, focal segmental glomerulosclerosis, nephrotic syndrome, pediatrics

## Abstract

**Introduction:**

Childhood-onset nephrotic syndrome has a variable clinical course. Improved predictive markers of long-term outcomes in children with nephrotic syndrome are needed. This study tests the association between baseline urinary epidermal growth factor (uEGF) excretion and longitudinal kidney function in children with nephrotic syndrome.

**Methods:**

The study evaluated 191 participants younger than 18 years enrolled in the Nephrotic Syndrome Study Network, including 118 with their first clinically indicated kidney biopsy (68 minimal change disease; 50 focal segmental glomerulosclerosis) and 73 with incident nephrotic syndrome without a biopsy. uEGF was measured at baseline for all participants and normalized by the urine creatinine (Cr) concentration. Renal epidermal growth factor (EGF) mRNA was measured in the tubular compartment microdissected from kidney biopsy cores from a subset of patients. Linear mixed models were used to test if baseline uEGF/Cr and EGF mRNA expression were associated with change in estimated glomerular filtration rate (eGFR) over time.

**Results:**

Higher uEGF/Cr at baseline was associated with slower eGFR decline during follow-up (median follow-up = 30 months). Halving of uEGF/Cr was associated with a decrease in eGFR slope of 2.0 ml/min per 1.73 m^2^ per year (*P* < 0.001) adjusted for age, race, diagnosis, baseline eGFR and proteinuria, and APOL1 genotype. In the biopsied subgroup, uEGF/Cr was correlated with EGF mRNA expression (*r* = 0.74; *P* < 0.001), but uEGF/Cr was retained over mRNA expression as the stronger predictor of eGFR slope after multivariable adjustment (decrease in eGFR slope of 1.7 ml/min per 1.73 m^2^ per year per log_2_ decrease in uEGF/Cr; *P* < 0.001).

**Conclusion:**

uEGF/Cr may be a useful noninvasive biomarker that can assist in predicting the long-term course of kidney function in children with incident nephrotic syndrome.

## Graphical abstract

**Figure:**
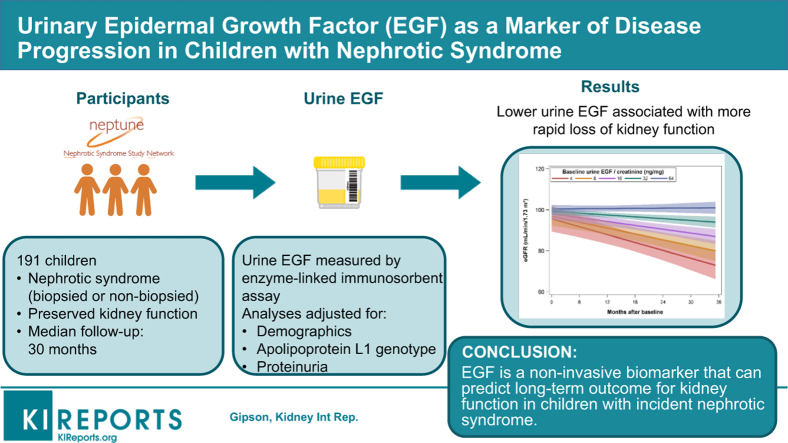

See Commentary on Page 383

Most cases of primary nephrotic syndrome in children are caused by 1 of 2 disorders: minimal change disease (MCD) or focal segmental glomerulosclerosis (FSGS).^1^ MCD is a generally favorable condition that presents with edema and proteinuria and is initially responsive to corticosteroid therapy in 75% to 90% of cases, depending on age of onset and ancestry.^2^ However, MCD subsequently follows a chronic course, and many children experience multiple relapses requiring second-line immunosuppressive therapy to control disease.^3^, ^4^, ^5^ Moreover, in addition to initial treatment resistance, an additional 20% develop late steroid resistance over time.^6^^,^^7^ In contrast, nearly 50% of patients with FSGS are unresponsive to current treatment regimens, either at disease onset or later in the course of their illness.^8^^,^^9^ Patients with treatment-resistant primary nephrotic syndrome, whether from MCD or FSGS, are at high risk of progression to end-stage kidney disease.^10^^,^^11^

Standard assessment of kidney biopsies can yield more than the histological diagnosis of MCD or FSGS. The severity of interstitial fibrosis and tubular atrophy (IFTA) has been associated with kidney disease progression.^12^ However, IFTA is not uniformly present throughout the kidney. Consequently, IFTA assessment from kidney biopsies is subject to sampling bias. Because young children with uncomplicated disease have a high likelihood of MCD, the standard of care in children younger than 12 years with new-onset nephrotic syndrome is to treat empirically with corticosteroids without routinely performing a kidney biopsy.^13^ Finally, there is considerable variability in clinical practice in the performance of a kidney biopsy later in the course of childhood nephrotic syndrome.

With or without a kidney biopsy, there is no clinical method to accurately distinguish at presentation the patients who are likely to have progressive loss of kidney function from those who will maintain kidney function long-term. The availability of a noninvasive test to improve prognostic evaluation for children with nephrotic syndrome could help guide decision making about further diagnostic assessment, therapy, and patient monitoring.

EGF is the prototypical peptide growth factor of the EGF/EGF receptor signaling pathway, which plays important roles in proliferation, differentiation, and migration of a variety of cell types, especially epithelial cells.^14^ In the kidney, EGF promotes tubular cell proliferation and has been linked to modulating the recovery from acute kidney injury.^15^^,^^16^ Our recent work has shown that EGF is inversely correlated with IFTA, that is, increased urinary excretion of EGF was associated with a lower degree of IFTA in renal biopsies obtained from adults with primary nephrotic syndrome or chronic kidney disease from a variety of causes.^17^ Further, the addition of EGF measures to demographic and clinical features improved the ability to predict progression of chronic kidney disease.^17^ Urinary EGF was recently reported to be associated with kidney disease progression in children with Alport syndrome^18^ and in a cohort of children predominantly with nonglomerular chronic kidney disease (CKD) (∼70% congenital anomalies of kidney and urinary tract).^19^ The utility of this urinary biomarker has not been assessed in children with nephrotic syndrome.

Therefore, we conducted the following study among a cohort of pediatric nephrotic syndrome patients to test the hypotheses that (i) urinary EGF excretion is associated with tubular compartment renal gene expression profiles of children with biopsied MCD and FSGS, and (ii) urinary EGF excretion is associated with cross-sectional and longitudinal kidney function in children with nephrotic syndrome, independent of demographic and clinical markers of disease.

## Methods

### Study Design

This longitudinal study used existing prospectively collected data and specimens from the National Institutes of Health–sponsored Nephrotic Syndrome Study Network (NEPTUNE).^20^ The primary outcome was the person-specific eGFR slope over follow-up, and the main predictor was uEGF at baseline.

### Study Data and Specimen Source

NEPTUNE, launched in 2010, is an ongoing prospective observational cohort study of children and adults with primary proteinuric kidney diseases.^20^ NEPTUNE data capture includes demographic data, clinical information of symptoms, diagnoses, physical examination, medications, laboratory values, biopsy tissue, patient-reported outcomes, health care utilization, procedures, and collection of urine and blood biosamples. The study includes 2 cohorts: an incident, nonbiopsy childhood-onset nephrotic syndrome (CO-NS) cohort and a biopsy cohort. The study visit schedule includes a baseline assessment within 45 days of onset of CO-NS nonbiopsied children or within 45 days of the kidney biopsy for biopsied children. Follow-up visits are conducted at months 1.5, 3, 8, and 12 (CO-NS), or every 4 months during the first year for the biopsy cohort. All enrolled participants are subsequently followed every 6 months. For this analysis, we excluded participants older than 18 years at the baseline visit; those with a biopsy diagnosis other than MCD, FSGS, or CO-NS; and those who did not have a baseline uEGF measurement. Institutional review board approval for this study was obtained at all participating sites with appropriate consent and assent forms.

Serum creatinine was measured in the central laboratory from specimens collected during study visits and enriched with data collected from local health records. GFR was estimated using the bedside serum creatinine–based pediatric estimating equation.^21^ Glomerular hyperfiltration was defined in this study as eGFR >120 ml/min per 1.73 m^2^. To reduce the effect of outlier measurements during periods of hyperfiltration wherein reduction in eGFR from hyperfiltration to normal range is considered improvement, all longitudinal eGFR measurements were Winsorized to 120 ml/min per 1.73 m^2^.^22^

Urine protein and creatinine were measured in the central laboratory from 24-hour or spot urine samples as available. Urine protein:creatinine ratios (UP:C) were expressed as mg/mg. These central measures were enriched by results collected from local medical records.

APOL1 risk alleles were genotyped directly via Sanger sequencing of the last 250 bases of exon 7.^23^ Participants were classified as having 2 (high risk) versus 0 or 1 risk alleles. No distinction was made between G1(rs73885319 and rs60910145) and the G2 indel (rs71785313). Participants who were nonblack were classified as having zero risk alleles based on the concept that APOL1 high-risk alleles are rarely present in individuals of European or Asian ancestry. African American participants who were genotyped but found to have 0 or 1 APOL1 risk alleles were similarly classified.

The local clinical kidney biopsy report was redacted of participant identifiers and submitted via the NEPTUNE electronic data capture system for review. Glass slides from the local clinical diagnostic kidney biopsy were submitted to the NEPTUNE Digital Pathology Repository for scanning and subsequent scoring by the NEPTUNE Core Pathology Committee.^24^

A single core of kidney biopsy tissue collected for research purposes during the initial local clinical kidney biopsy was preserved in RNAlater and submitted to NEPTUNE laboratories at the University of Michigan. Biopsy tissue was microdissected into tubular and glomerular compartments and renal gene expression data were generated using Affymetrix GeneChip Human Genome ST2.1.^23^ The details of tissue harvesting, microdissection, RNA isolation, reverse transcription, linear amplification, and target preparation followed published strategies.^25^^,^^26^ The fragmentation, hybridization, staining, and imaging were performed according to the Affymetrix Expression Analysis Technical Manual. The .CEL files were processed and normalized by the Robust MultiArray method and annotated with the Human Entrez Genes custom Chip Description File (http://brainarray.mbni.med.umich.edu)*.*^27^ Normalized expression value data were log_2_ transformed and batch corrected using ComBat.^28^

uEGF was assessed in duplicate from urine collected at study visits using enzyme-linked immunosorbent assay (R&D Systems, Inc., Minneapolis, MN) and optimized in the laboratory as previously reported.^17^ uEGF was normalized for urine creatinine concentration (uEGF/Cr ng/mg) to adjust for differences in urine concentration between participants.

### Data Analyses

Descriptive analyses were conducted on all participants who had a baseline uEGF measurement using frequencies and percentages for categorical variables and medians and interquartile ranges for continuous variables. Comparisons across disease categories were made using χ^2^ tests for categorical variables and Kruskal-Wallis tests for continuous variables. To reduce skewness, a log_2_ transformation was applied to the mean of the uEGF/Cr duplicate values. Pearson correlations were calculated to examine potential associations between log_2_(uEGF/Cr) and log_2_(renal *EGF* mRNA expression) when both samples were collected within a 7-day interval. All eGFR values were Winsorized (capped) at 120 ml/min per 1.73 m^2^.

Longitudinal linear mixed effects models with random intercepts and slopes were used to predict the outcome of eGFR. Analyses began at the earliest time of study data collection: time of biopsy for the MCD and FSGS cohorts; time of nephrotic syndrome onset for the nonbiopsied CO-NS cohort. Covariates of primary interest were time from biopsy, baseline uEGF/Cr, and an interaction term between time and uEGF/Cr to test whether trajectories of eGFR differ by uEGF/Cr at baseline. For example, a significant main effect for uEGF/Cr would yield parallel eGFR slopes over follow-up, differing by a linear function of uEGF/Cr at baseline; a significant interaction would indicate nonparallel (fan-shaped) slopes in eGFR over time, reflecting both a shift up or down and a change in slope as a linear function of baseline uEGF/Cr. Other variables included in the models were baseline values of age, prior disease duration, prior therapy, UP:C, and eGFR, as well as sex, race, ethnicity, kidney disease diagnosis, and APOL1 genotype. Interaction terms between time and each predictor of interest were tested as potential predictors of eGFR slope, using Wald tests to assess significance. All variables were included in a backwards multivariable model selection. The backwards selection process ensured the model was hierarchically well-formulated. First, nonsignificant interactions with time were removed in descending order of *P* value. Then main effects underwent backwards selection, but any main effects terms part of a statistically significant interaction with time were retained in the final models. In addition, age was included in all models (regardless of significance) to account for the known inverse relationship between EGF and age.^29^ Although Wald tests are standard for routine assessment of significance, the variable of primary interest, uEGF/Cr, was also tested by likelihood ratio tests, which are generally more accurate.

A separate analysis was conducted in the biopsied participants alone to further examine whether or not renal biopsy IFTA, global sclerosis, or *EGF* RNA expression were predictors of eGFR slope using the same longitudinal mixed models and backwards selection approach. Sensitivity analyses were conducted refitting final models using age-normalized values of uEGF/Cr. Normalization was done using median-interquartile range age-normalization of uEGF/Cr based on data from healthy children reported by Meybosch *et al.*^29^^,^^30^ All analyses were conducted using SAS v9.4 (SAS Institute, Inc., Cary, NC). A significance level of 0.05 and 2-sided testing were used throughout; 95% confidence intervals (CIs) were reported.

## Results

**Figure 1:**
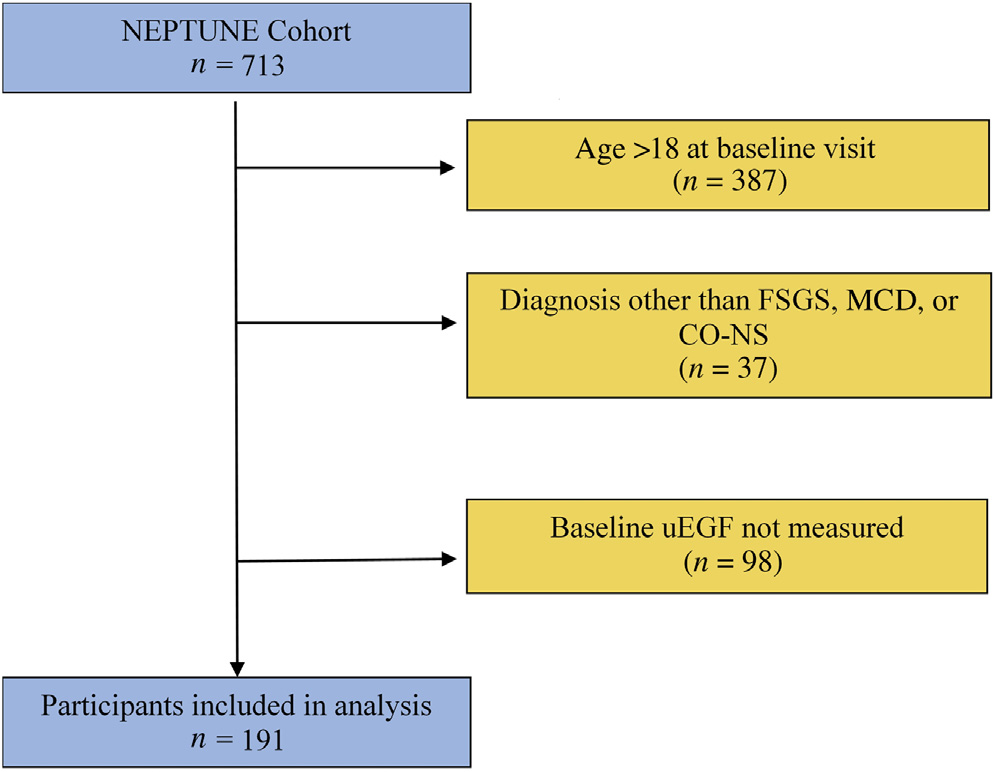
Flow diagram of included participants. CO-NS, childhood-onset nephrotic syndrome not biopsied; FSGS, focal segmental glomerulosclerosis; MCD, minimal change disease; uEGF, urinary epidermal growth factor.

As of May 2019, there were 713 participants enrolled in NEPTUNE, 326 of whom were <18 years old at the baseline visit (Figure 1). Of these, 37 were excluded because they had a diagnosis other than FSGS, MCD, or CO-NS, and a further 98 did not have a baseline uEGF/Cr measurement, leaving 191 participants for analysis. Baseline characteristics of these participants are shown by diagnosis in Table 1. Comparing FSGS, MCD, and CO-NS, respectively, median age varied by cohort (13, 11, and 4 years, *P* < 0.001), as did median baseline eGFR (90, 105, and 139 ml/min per 1.73 m^2^, *P* < 0.001) and median uEGF/Cr (24.9, 39.9, and 71.4 ng/mg, *P* < 0.001). At baseline uEGF/Cr collection, 37% of participants had nephrotic range proteinuria (UP:C ≥3.0 g/g), 27% sub-nephrotic, but active, proteinuria (UP:C <3.0 g/g and UP:C ≥0.3 g/g), and 35% were in a complete remission of proteinuria (UP:C <0.3 g/g). However, there was no correlation between baseline uEGF/Cr and UP:C (*r* = −0.07, *P* = 0.34). There was also no association between uEGF receptor/Cr and steroid response pattern, both initially and after 1 year (Supplementary Figure S1).

**Table 1:** Comparison of baseline characteristics of pediatric Nephrotic Syndrome Study Network participants with a baseline uEGF/Cr measurement by diagnosis (*n* = 191)

Characteristic	All children (*n* = 191)	CO-NS (*n* = 73)	MCD (*n* = 68)	FSGS (*n* = 50)	*P* value
Age at baseline (yr)	7 (4, 13)	4 (3, 7)	11 (6, 14)	13 (6, 15)	<0.001
Sex (female)	84 (44)	36 (49)	26 (38)	22 (44)	0.42
Race					0.07
White	80 (42)	36 (49)	29 (43)	15 (30)	
Black	62 (32)	17 (23)	20 (29)	25 (50)	
Asian	24 (13)	9 (12)	12 (18)	3 (6)	
Native American	7 (4)	2 (3)	3 (4)	2 (4)	
Not reported	18 (9)	9 (12)	4 (6)	5 (10)	
Hispanic or Latino	38 (20)	12 (16)	13 (19)	13 (26)	0.75
Kidney disease duration (mo)	1 (0, 7)	0 (0, 1)	8 (1, 30)	2 (1, 20)	<0.001
Received treatment before enrollment	65 (34)	0 (0)	38 (56)	27 (54)	<0.001
eGFR at baseline (ml/min per 1.73 m^2^)	108 (89, 135)	139 (100, 193)	105 (90, 125)	90 (74, 114)	<0.001
Hyperfiltration^a^ at baseline	71 (37)	42 (58)	21 (31)	8 (16)	<0.001
UP:C at baseline (g/g)	5.9 (1.4, 10.6)	7.3 (0.3, 12.6)	5.6 (1.5, 10.3)	3.6 (1.7, 8.2)	0.56
Interstitial fibrosis (%)	1 (0, 4)	–	0 (0, 2)	3 (0, 10)	0.002
APOL1 (2 risk alleles)	19 (10)	1 (1)	3 (4)	15 (30)	<0.001
uEGF/Cr (ng/mg)	42.0 (27.2, 71.6)	71.4 (40.0, 91.3)	39.9 (27.3, 55.6)	24.9 (11.4, 41.2)	<0.001
Log_2_- EGF RNA expression	7.3 (6.6, 7.6)	–	7.5 (7.3, 7.8)	6.9 (6.4, 7.3)	0.005
Months of follow-up	30 (17, 52)	19 (10, 29)	44 (25, 54)	50 (27, 56)	<0.001
eGFR slope (ml/min per 1.73 m^2^/yr)^b^	−2.9 [−4.4 to −1.4]	1.0 [−2.3 to 4.2]	−2.3 [−4.5 to −0.1]	−5.4 [−7.7 to −3.0]	0.007

CO-NS, childhood-onset nephrotic syndrome, not biopsied; EGF, epidermal growth factor; eGFR, estimated glomerular filtration rate; FSGS, focal segmental glomerulosclerosis; IQR, interquartile range; MCD, minimal change disease; uEGF/Cr, urinary EGF:creatinine ratio; UP:C, urine protein:creatinine ratio

eGFR slope estimates were derived from a linear mixed-effects model and values reported are mean and 95% confidence intervals. Other continuous variables are reported as median (interquartile range) and use a Kruskal-Wallis test for comparison; categorical variables are reported as n (%) and use a χ^2^ test for comparison

a eGFR>120 ml/min per 1.73m^2^.

b Derived from linear mixed effects model.

**Figure 2:**
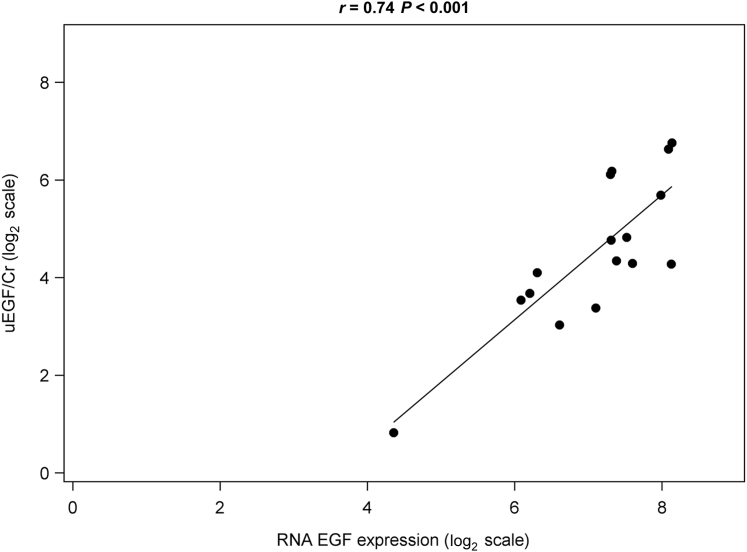
Epidermal growth factor **(**EGF) mRNA expression correlates with urinary EGF (uEGF)/creatinine (Cr) among biopsied pediatric Nephrotic Syndrome Study Network participants. Urine collection for uEGF/Cr occurred within 7 days of biopsy. Data were plotted only for participants with EGF mRNA RNA expression data available (*n* = 16).

Urinary EGF/Cr was strongly correlated with renal *EGF* mRNA expression (both log_2_ transformed) in the 16 participants with matching samples within 1 week of biopsy (Figure 2, *r* = 0.74, *P* < 0.001). In addition, uEGF/Cr was moderately correlated with baseline eGFR (Supplementary Figure S2, *r* = 0.49, *P* < 0.001).

### Regression Analyses

The adjusted relationship between uEGF/Cr and eGFR over time was assessed using multivariable linear mixed effects models developed with backward selection (Table 2, model 1). Baseline values of age, eGFR, UP:C, and uEGF/Cr, as well as race, diagnosis, and APOL1 genotype were all significant predictors of eGFR and included in the final model. Significant interactions with time indicate that different levels of a variable are associated with different values of the slope of eGFR over time. For example, doubling of uEGF/Cr was associated with a 2.0 ml/min per 1.73 m^2^/year increase in eGFR slope (95% CI: 1.1–2.9; *P* < 0.001). Conversely, halving of EGF/Cr was associated with a 2.0 ml/min per 1.73 m^2^ decrease in eGFR slope. In addition, a 1-log_e_ increase in UP:C was associated with a steeper decline in eGFR by −1.1 ml/min per 1.73 m^2^/year (95% CI: −1.6 to −0.5; *P* < 0.001). Native American participants also had more rapid eGFR decline compared with white participants (−5.6 ml/min per 1.73 m^2^/year; 95% CI: −9.6 to −1.7; *P* = 0.01), as did FSGS compared with CO-NS participants (−4.4 ml/min per 1.73 m^2^/year; 95% CI: −7.6 to −1.2; *P* = 0.01). Significant main effects, in the absence of a significant interaction with time, indicate that a variable was associated with higher or lower eGFR, but that it was not associated with change in slope of eGFR over time. For example, high-risk APOL1 genotype was associated with lower eGFR (main effect: −15.2 ml/min per 1.73 m^2^; 95% CI: −19.1 to −11.3), but not a significant interaction between genotype and eGFR slope. Model 1 was refit after removing uEGF/Cr (Table 2, model 2). A likelihood ratio test demonstrated an improvement in fit after including uEGF/Cr (Table 2: model 1 vs. model 2, *P* < 0.001).

**Table 2:** Adjusted longitudinal linear mixed-effects models of eGFR over time

Outcome = eGFR (ml/min per 1.73 m^2^)	Model 1 (with uEGF/Cr)		Model 2 (without uEGF/Cr)	
	Estimate [95% CI]	*P* value	Estimate [95% CI]	*P* value
Main effects				
Intercept	52.1	–	51.4	*–*
Follow-up time (per yr)	−13.0 [−19.4 to −6.7]	<0.001	−0.3 [−2.7 to 2.0]	0.80
Age (per yr older)	−0.1 [−0.5 to 0.3]	0.56	−0.2 [−0.6 to 0.2]	0.35
Race				
Asian vs. White	0.5 [−3.7 to 4.7]	0.80	1.6 [−2.7 to 5.8]	0.48
Black vs. White	0.6 [−3.3 to 4.5]	0.76	0.7 [−3.1 to 4.4]	0.73
Native American vs. White	3.7 [−5.1 to 12.4]	0.41	4.4 [−4.5 to 13.2]	0.34
Diagnosis				
MCD vs. CO-NS	4.7 [0.6–8.7]	0.02	4.3 [0.2–8.5]	0.04
FSGS vs. CO-NS	−3.4 [−8.2 to 1.5]	0.17	−4.2 [−9.0 to 0.7]	0.09
Winsorized eGFR at baseline	0.4 [0.3–0.4]	<0.001	0.5 [0.4–0.5]	<0.001
log UP:C at baseline	−0.4 [−1.4 to 0.6]	0.46	−0.5 [−1.5 to 0.6]	0.36
APOL1 (2 risk alleles vs. 0/1)	−15.2 [−19.1 to −11.3]	<0.001	−14.0 [−18.0 to −10.1]	<0.001
log_2_ uEGF/Cr at baseline	1.2 [−0.6 to 3.0]	0.19	–	–
Interaction with time (eGFR slope per year) (ml/min per 1.73 m^2^/yr)				
Age (per yr older)	0.3 [0.0–0.5]	0.02	−0.0 [−0.2 to 0.2]	0.99
Race				
Asian vs. White	−3.4 [−5.5 to −1.2]	0.002	−3.9 [−6.1 to −1.7]	<0.001
Black vs. White	0.5 [−1.5 to 2.5]	0.64	−2.0 [−3.8 to −0.2]	0.03
Native American vs. White	−5.6 [−9.6 to −1.7]	0.01	−5.0 [−9.1 to −1.0]	0.01
Diagnosis				
MCD vs. CO-NS	−2.9 [−5.9 to 0.1]	0.06	−2.7 [−5.8 to 0.3]	0.08
FSGS vs. CO-NS	−4.4 [−7.6 to −1.2]	0.01	−4.8 [−8.1 to −1.6]	0.003
log UP:C at baseline	−1.1 [−1.6 to −0.5]	<0.001	−1.0 [−1.5 to −0.4]	<0.001
log_2_ uEGF/Cr at baseline	2.0 [1.1–2.9]	<0.001	–	–

CI, confidence interval; CO-NS, childhood-onset nephrotic syndrome, not biopsied; EGF, epidermal growth factor; eGFR, estimated glomerular filtration rate (ml/min per 1.73 m^2^); FSGS, focal segmental glomerulosclerosis; MCD, minimal change disease; uEGF/Cr, urinary EGF:creatinine ratio (ng/mg); UP:C, urine protein:creatinine ratio (g/g).

Results from all pediatric Nephrotic Syndrome Study Network participants with baseline uEGF/Cr data available (*n* = 191 participants, 1553 observations). Likelihood ratio test comparing model 1 with model 2: *P* < 0.001. Variables tested for inclusion in the model are sex, ethnicity, kidney disease duration at baseline, and prior therapy at baseline.

**Figure 3:**
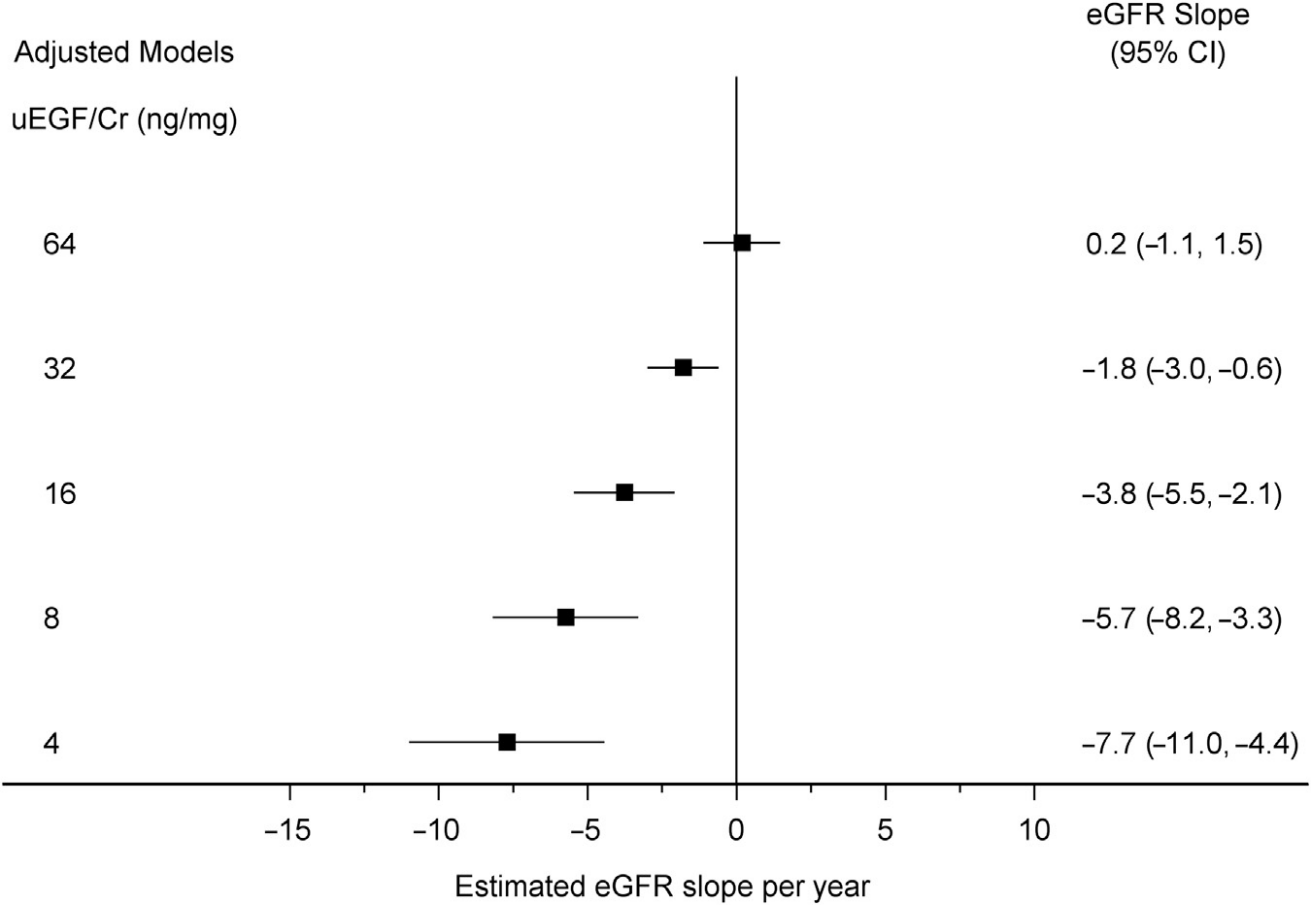
The association between baseline urinary epidermal growth factor/creatinine (uEGF/Cr) and estimated glomerular filtration rate (eGFR) over time. Results from adjusted linear mixed effects models among all pediatric Nephrotic Syndrome Study Network participants with baseline uEGF/Cr data available (*n* = 191 participants, 1553 observations). eGFR values Winsorized (capped) to 120. CI, confidence interval.

**Figure 4:**
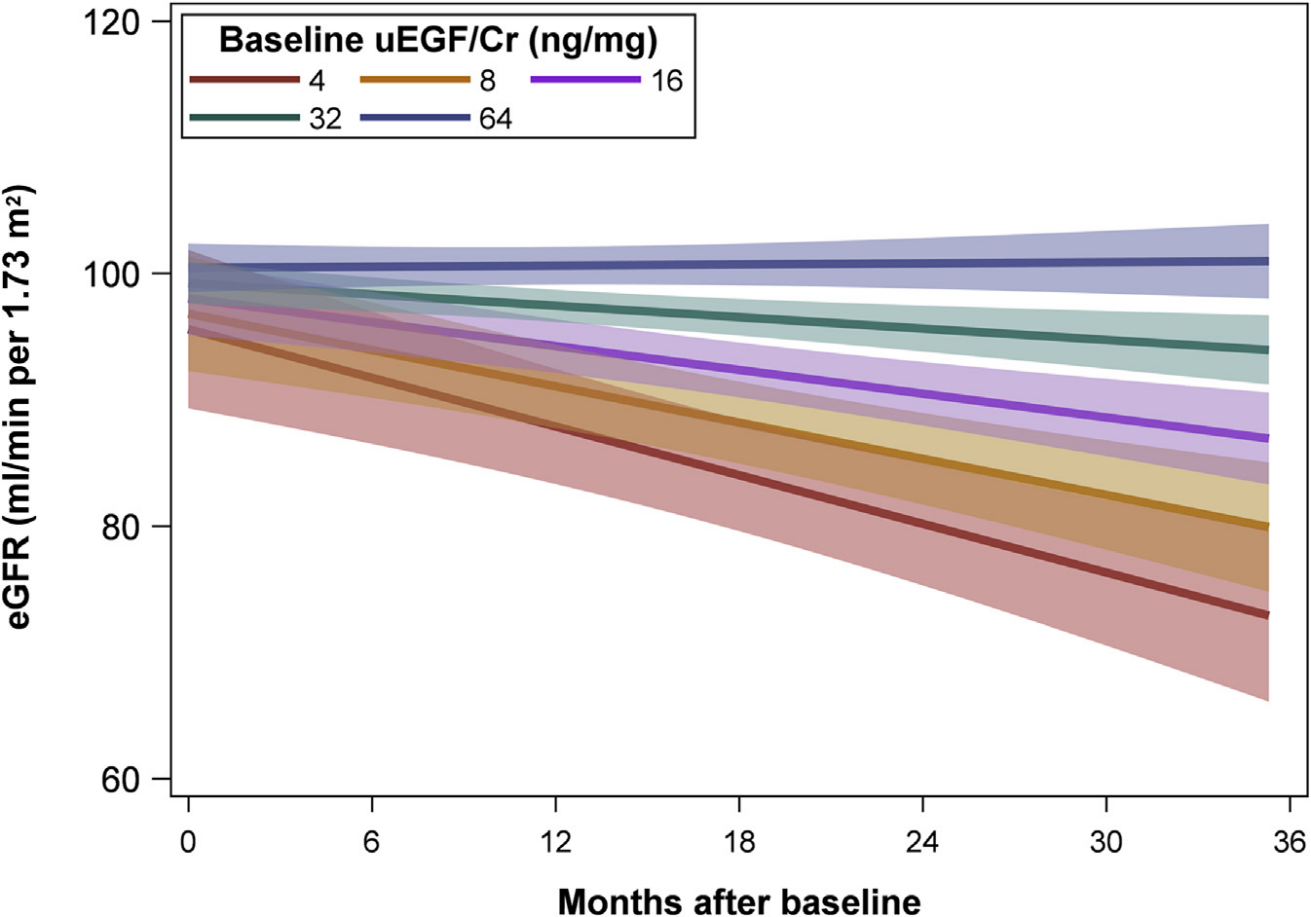
The association between baseline urinary epidermal growth factor/creatinine (uEGF/Cr) and estimated glomerular filtration rate (eGFR) over time. Results from adjusted linear mixed effects models among all pediatric Nephrotic Syndrome Study Network participants with baseline uEGF/Cr data available (*n* = 191 participants, 1553 observations). eGFR values Winsorized (capped) at 120 ml/min per 1.73 m^2^. Values shown are regression estimates and 95% confidence intervals.

Figure 3 shows the eGFR slope for different values of uEGF/Cr based on model 1 in Table 2, which includes both biopsied and nonbiopsied participants. Participants with a lower uEGF/Cr had a more rapid decline in eGFR. For example, holding constant other variables in the model, a participant with a baseline uEGF/Cr of 32 ng/mg had an estimated slope of −1.8 ml/min per 1.73 m^2^/year (95% CI: −3.0 vs. −0.6), whereas a participant with a baseline uEGF/Cr of 16 ng/mg had an estimated slope of −3.8 ml/min per 1.73 m^2^/year (95% CI: −5.5 to −2.1). Model derived estimates of eGFR over time with 95% CIs are also shown in Figure 4.

Table 3 shows an analogous multivariable model of eGFR restricted to biopsied participants only. uEGF/Cr was a significant predictor of change in eGFR over time among these participants with coefficients of similar magnitude reported in model 1 of Table 2 versus Table 3. Among biopsied participants, a doubling of uEGF/Cr was associated with a 1.7 ml/min per 1.73 m^2^/year improvement in eGFR slope (95% CI: 0.6–2.8; *P* < 0.001). This model was also adjusted for biopsy characteristics (diagnosis, interstitial fibrosis, and global sclerosis), which were also independently associated with eGFR. As seen previously in Table 2, adding uEGF/Cr to the model improved model fit (Table 3: model 1 vs. model 2, *P* < 0.001).

**Table 3:** Adjusted longitudinal linear mixed-effects models of eGFR over time

Outcome = eGFR (ml/min per 1.73 m^2^)	Model 1 (with uEGF/Cr)		Model 2 (without uEGF/Cr)	
	Estimate [95% CI]	*P* value	Estimate [95% CI]	*P* value
Main effects				
Intercept	33.3	–	31.6	*–*
Follow-up time (per yr)	−15.0 [−22.7 to −7.3]	<0.001	−3.9 [−7.1 to −0.6]	0.02
Age (per yr older)	−0.1 [−0.7 to 0.5]	0.79	−0.1 [−0.7 to 0.5]	0.74
Race				
Asian vs. White	−2.3 [−9.4 to 4.8]	0.52	−0.3 [−7.4 to 6.9]	0.94
Black vs. White	2.6 [−3.5 to 8.7]	0.40	2.7 [−3.2 to 8.5]	0.37
Native American vs. White	8.9 [−4.1 to 21.8]	0.18	9.9 [−3.3 to 23.1]	0.14
Diagnosis				
MCD vs. FSGS	7.7 [4.3–11.0]	<0.001	8.7 [5.2–12.1]	<0.001
Winsorized eGFR at baseline	0.6 [0.5–0.7]	<0.001	0.7 [0.6–0.7]	<0.001
log UP:C at baseline	1.0 [−0.8 to 2.9]	0.28	0.9 [−1.0 to 2.8]	0.33
APOL1 (2 risk alleles vs. 0/1)	−9.5 [−14.6 to −4.3]	<0.001	−6.7 [−11.8 to −1.5]	0.01
log Interstitial fibrosis (%)	−1.2 [−2.1 to −0.2]	0.02	−1.5 [−2.4 to −0.5]	0.003
Log global sclerosis (%)	0.4 [−0.8 to 1.6]	0.52	0.5 [−0.7 to 1.8]	0.40
log_2_ uEGF/Cr at baseline	0.9 [−1.3 to 3.2]	0.41	–	–
Interaction with time (eGFR slope per year) (ml/min per 1.73 m^2^/yr)				
Age	0.4 [0.1–0.7]	0.003	0.2 [−0.1 to 0.4]	0.15
Race				
Asian vs. White	−2.5 [−5.4 to 0.4]	0.08	−3.4 [−6.3 to −0.4]	0.03
Black vs. White	0.1 [−2.6 to 2.7]	0.97	−2.2 [−4.6 to 0.1]	0.07
Native American vs. White	−10.0 [−14.9 to −5.2]	<0.001	−10.1 [−15.1 to −5.1]	<0.001
log UP:C at baseline	−1.4 [−2.2 to −0.7]	<0.001	−1.3 [−2.1 to −0.5]	0.001
Log global sclerosis (%)	−0.9 [−1.3 to −0.4]	<0.001	−0.8 [−1.3 to −0.4]	0.001
log2 uEGF/Cr at baseline	1.7 [0.6–2.8]	0.002	–	–

CI, confidence interval; eGFR, estimated glomerular filtration rate (ml/min per 1.73 m^2^); EGF, epidermal growth factor; FSGS, focal segmental glomerulosclerosis; MCD, minimal change disease; uEGF/Cr, urinary EGF:creatinine ratio (ng/mg); UP:C, urine protein: creatinine ratio (g/g).

Results restricted to pediatric participants with kidney biopsy (*n* = 118 participants, 1164 observations). Likelihood ratio test comparing model 1 with model 2: *P* < 0.001. Variables tested for inclusion in the model are sex, ethnicity, kidney disease duration at baseline, prior therapy at baseline, and EGF RNA expression.

Sensitivity analyses were conducted using age-normalized uEGF/Cr instead of log_2_(uEGF/Cr). These analyses refit final models both from all participants (Supplementary Table S1) and from biopsied participants only (Supplementary Table S2) and confirm the preceding findings: lower uEGF/Cr per age was associated with more rapid eGFR decline after adjusting for age, race, biopsy characteristics, and baseline eGFR and UP:C and APOL1 genotype.

## Discussion

This study was conducted to assess the potential value of uEGF measurement as a prognostic marker for kidney function loss in children with nephrotic syndrome. The study population included children at initial presentation of nephrotic syndrome and children undergoing an initial kidney biopsy for clinical indications. This study found that the addition of uEGF to the traditional predictive demographic and clinical laboratory findings improved the precision of eGFR slope estimates in children with nephrotic syndrome.

EGF is a member of the epidermal growth factor family and acts through the EGF receptor. Binding of EGF to EGF receptors induces release of calcium from intracellular stores, activation of signaling pathways, cell differentiation, and cell repair. Exogenous EGF has been shown to enhance renal tubular cell regeneration and accelerate the recovery of the kidney function in a rodent model of acute kidney injury.^15^ Our recent work using integrated machine learning and kidney biopsy transcriptome-driven approach identified and validated uEGF as a kidney tubular cell–specific biomarker representing functional tubular mass and regeneration potential.^17^ uEGF is positively correlated with eGFR in patients with CKD and inversely correlated with tubular atrophy and interstitial fibrosis.^15^^,^^17^ As plasma EGF is minimal and the kidney is the organ expressing the most abundant EGF mRNA among a panel of 79 human tissue/cells,^17^^,^^31^ the accepted origin of uEGF is kidney tubular cells. uEGF is correlated with intrarenal *EGF* mRNA level in adult patients with CKD.^17^^,^^18^ Because of kidney-specificity for production, uEGF level is less confounded compared with other clinical markers that may have non-kidney origins (e.g., serum creatinine from muscle mass or proteinuria from systemic light chain proteins in circulation).

Our multivariable modeling among biopsied patients showed that uEGF was superior to EGF mRNA expression in predicting eGFR loss. One explanation could be that due to the focal nature of kidney injury, measured *EGF* intrarenal mRNA expression is affected by variability in biopsy specimen sampling, whereas uEGF is excreted by tubular cells from the entire kidney and therefore more accurately reflects global kidney function.

Two recent publications reported that a lower baseline uEGF level is associated with kidney disease progression in children with Alport Syndrome^18^ and children with CKD (primarily congenital anomalies of kidney and urinary tract).^19^ Here we demonstrated that lower uEGF level is significantly associated with a more rapid eGFR decline in children with nephrotic syndrome, independent of proteinuria control or steroid response pattern. Our finding is consistent with these 2 studies despite different etiologies of CKD, and provides additional evidence supporting the prognostic value of uEGF in children with kidney disease. A noninvasive urinary biomarker is particularly valuable for assessing prognosis in children. If these results are confirmed, the addition of uEGF measurement to the diagnostic evaluation of a child presenting with nephrotic syndrome may improve the precision with which prognosis is assessed. For example, a low uEGF value suggests that the child has reduced intact kidney parenchyma and reduced repair capacity and may therefore be at an increased risk for eGFR decline. This information may prompt earlier performance of a kidney biopsy to assess the integrity of the kidney and help the family and clinician develop a management plan that aligns with progression risk. These approaches could include early use of renin-angiotensin system blockade, intensive blood pressure control to the 50th percentile target,^32^ and anticipatory guidance of the need for second-line therapies should the child be resistant to this treatment.

The current study and previous findings implicate low uEGF as a predictive marker of kidney disease progression.^17^ Rat models demonstrate that exogenous EGF can enhance renal tubular cell regeneration and repair.^15^ Therefore, we hypothesize that stabilization of EGF and EGF receptor activation in a cell-selective context in the kidney tubules may ameliorate kidney disease progression. However, given the long-established oncogenic role that EGF receptor activation plays in human cancers, and dramatic responses in patients with cancer to therapies that inhibit oncogenic EGF receptor activation,^33^ using therapies around activation or reactivation of ubiquitous EGF expression for treatment of kidney disease has the potential for off-target effects. Properly controlled increased expression of EGF specifically in tubular epithelial cells may offer a feasible alternative.

### Study Limitations

The findings in this report are based on a single determination of uEGF excretion. This study requires replication with a similar cohort of children with nephrotic syndrome and long-term observation of both uEGF excretion and kidney disease outcomes. In addition, our study imputed APOL1 for white, Asian, and Native American participants as low-risk APOL1 genotype. Finally, eGFR was based on serum Cr, as cystatin C was not available from all study visits.

A limitation to clinical implementation of these results is the absence of a rigorously derived reference range for uEGF/Cr by age and sex. EGF is known be inversely correlated with age. Although these results showed a significant impact of EGF on disease trajectory after accounting for age, accurate clinical interpretation of EGF at the patient level will require standardization by age. Future research should derive reference ranges for EGF by age and sex.

In conclusion, uEGF/Cr may be a useful noninvasive biomarker that can assist in predicting long-term kidney function in children with incident nephrotic syndrome. The published evidence from children and adults with other types of kidney diseases is bolstered by the demonstrated value of this biomarker, with or without a kidney biopsy, in children with nephrotic syndrome.

## Disclosure

JPT and DSG have research funding through the University of Michigan with Complexa Inc., Retrophin Inc., and Goldfinch Bio (DSG additionally has funding through the University of Michigan with Bristol-Meyers Squibb; JPT additional has fundingthrough the University of Michigan with Vertex Phamaceuticals and Pfizer Inc.). SE has research funding through the University of Michigan with AstraZeneca PLC, Eli Lilly and Company, Novo Nordisk A/S, Gilead Sciences, Inc., and Moderna, Inc., and owns shares of Gilead Sciences, Inc., Johnson & Johnson, ThermoFisher Scientific, Inc, and AbbVie, Inc. TS has received research support from Bristol-Myers Squibb, Mallinckrodt Pharmaceuticals, Goldfinch, and Retrophin, Inc. HT is a consultant to Otsuka (Chair, DMC) and Chemocentryx (DMC) and a consultant to Goldfinch and Retrophin (in contract through New York University). KJR is a site principal investigator for a Complexa study and Advicienne study unrelated to these data. MK receives research support through the University of Michigan with AstraZeneca PLC, Eli Lilly and Company, Novo Nordisk A/S, Gilead Sciences, Inc., Jansen, Angion, Certa, and Moderna, Inc. WJ and MK have a patent pending on biomarkers for chronic kidney disease (CKD) progression (encompassing urinary epidermal growth factor as a biomarker of CKD progression). All other disclosures are unrelated to these data. All the other authors declared no competing interests.

## Supplementary Material

Supplementary File (Word)
