# Supplementary material for: Urinary Epidermal Growth Factor as a Marker of Disease Progression in Children With Nephrotic Syndrome
Source: Kidney Int Rep. 2019 Dec 5;5(4):414–25. doi: 10.1016/j.ekir.2019.11.018 (PMC7136430; doi:10.1016/j.ekir.2019.11.018)
Supplement: Supplementary File (Word) [file mmc1.docx]

**Supplemental Material: Table of Contents**

[Supplemental Figure 1. No relationship between uEGF/Cr and (A) initial steroid response pattern or (B) response pattern after 1 year 2](#_Toc24616177)

[Supplemental Figure 2. Baseline eGFR correlates with uEGF/Cr among pediatric NEPTUNE participants (n=191) 3](#_Toc24616178)

[Supplemental Table 1. Sensitivity analysis of age-normalized uEGF/Cr. Adjusted longitudinal linear mixed-effects models of eGFR over time. Results from all pediatric NEPTUNE participants with baseline uEGF/Cr data available (n=191 participants, 1,553 observations). Likelihood ratio test comparing Model 1 to Model 2: p < 0.001 4](#_Toc18058112)

[Supplemental Table 2. Sensitivity analysis of age-normalized uEGF/Cr. Adjusted longitudinal linear mixed-effects models of eGFR over time. Results restricted to pediatric participants with kidney biopsy. (n=118 participants, 1,164 observations). Likelihood ratio test comparing Model 1 to Model 2: p <0.001 5](#_Toc18058113)

Supplemental Figure 1. No relationship between uEGF/Cr and (A) initial steroid response pattern or (B) response pattern after 1 year

| **(A)** | **(B)** |
| --- | --- |
| 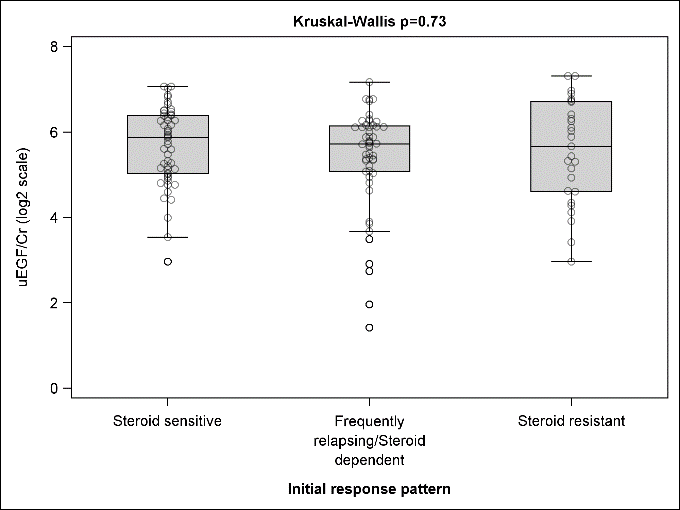 | 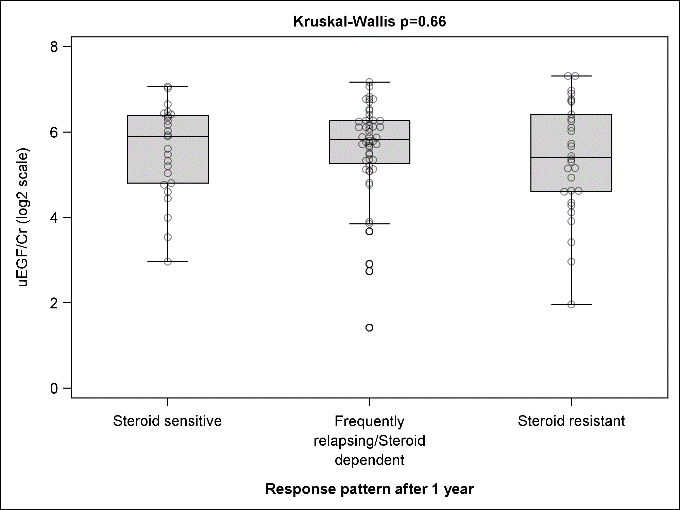 |

Supplemental Figure 2. Baseline eGFR correlates with uEGF/Cr among pediatric NEPTUNE participants (n=191)


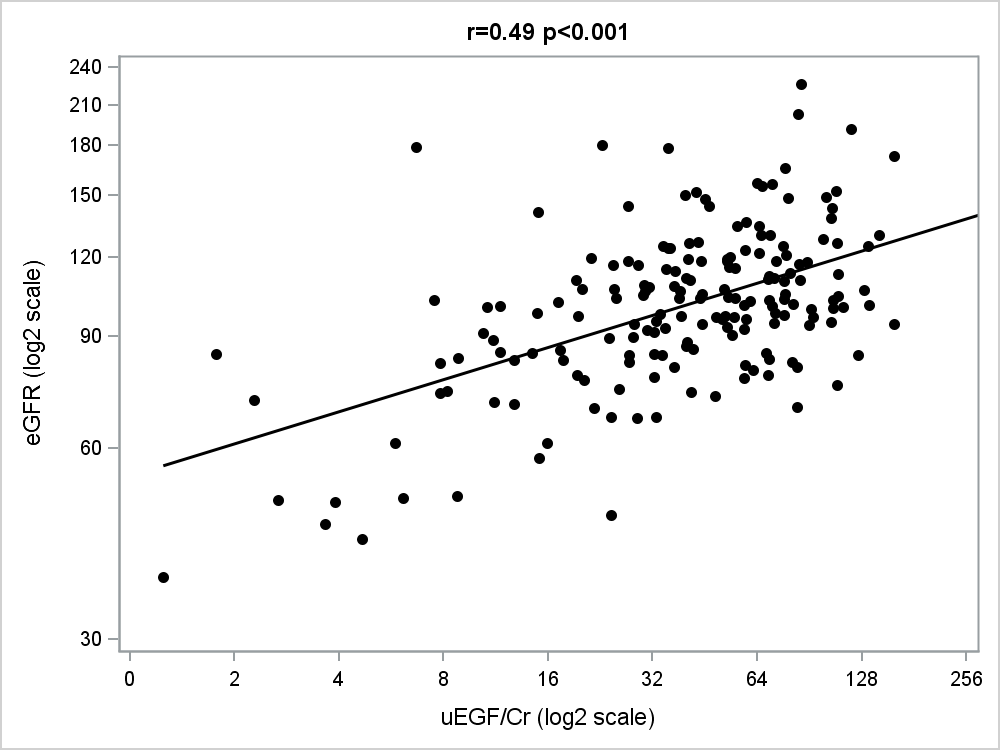


Abbreviations: eGFR=estimated glomerular filtration rate mL/min/1.73m^2^); EGF=epidermal growth factor; uEGF/Cr=urinary EGF: creatinine ratio (ng/mg)

Supplemental Table 1. Sensitivity analysis of age-normalized uEGF/Cr. Adjusted longitudinal linear mixed-effects models of eGFR over time. Results from all pediatric NEPTUNE participants with baseline uEGF/Cr data available (n=191 participants, 1,553 observations). Likelihood ratio test comparing Model 1 to Model 2: p < 0.001

| Outcome = eGFR (mL/min/1.73m^2^) | **Model 1**  **(with uEGF/Cr)** | | **Model 2**  **(without uEGF/Cr)** | |
| --- | --- | --- | --- | --- |
|  | Estimate  [95% CI] | p-value | Estimate  [95% CI] | p-value |
| Main effects |  |  |  |  |
| Intercept | 55.0 | --- | 50.5 | *---* |
| Follow-up time (per year) | 1.9 [-0.6, 4.5] | 0.13 | -0.3 [-2.6, 2.0] | 0.80 |
| Age (per year older) | -0.2 [-0.6, 0.2] | 0.31 | -0.2 [-0.6, 0.2] | 0.35 |
| Race |  |  |  |  |
| Asian vs. White | 1.8 [-2.4, 6.0] | 0.41 | 1.6 [-2.7, 5.8] | 0.48 |
| Black vs. White | 0.2 [-3.7, 4.1] | 0.92 | 0.7 [-3.1, 4.4] | 0.73 |
| Native American vs. White | 3.1 [-5.7, 11.8] | 0.49 | 4.4 [-4.5, 13.2] | 0.34 |
| Diagnosis |  |  |  |  |
| MCD vs. CO-NS | 3.0 [-1.9, 8.0] | 0.22 | 4.2 [-0.7, 9.0] | 0.09 |
| FSGS vs. CO-NS | 7.1 [3.1, 11.0] | <0.001 | 8.5 [4.6, 12.4] | <0.001 |
| Winsorized eGFR at baseline | 0.4 [0.4, 0.5] | <0.001 | 0.5 [0.4, 0.5] | <0.001 |
| log UP:C at baseline | -0.6 [-1.6, 0.5] | 0.29 | -0.5 [-1.5, 0.6] | 0.36 |
| APOL1 (2 risk alleles vs 0/1) | -15.5 [-19.3, -11.6] | <0.001 | -14.0 [-18.0, -10.1] | <0.001 |
| log2 uEGF/Cr at baseline | 1.4 [-4.8, 7.6] | 0.66 | --- | --- |
| Interaction with time (eGFR slope per year) (mL/min/1.73m2/year) |  |  |  |  |
| Age | -0.1 [-0.3, 0.1] | 0.41 | -0.0 [-0.2, 0.2] | 0.99 |
| Race |  |  |  |  |
| Asian vs. White | -3.2 [-5.3, -1.1] | 0.003 | -3.9 [-6.1, -1.7] | <0.001 |
| Black vs. White | 0.3 [-1.7, 2.3] | 0.77 | -2.0 [-3.8, -0.2] | 0.03 |
| Native American vs. White | -4.5 [-8.4, -0.5] | 0.03 | -5.0 [-9.1, -1.0] | 0.01 |
| Diagnosis |  |  |  |  |
| MCD vs. CO-NS | 4.4 [1.2, 7.6] | 0.01 | 4.8 [1.6, 8.1] | 0.003 |
| FSGS vs. CO-NS | 1.9 [0.2, 3.6] | 0.03 | 2.1 [0.4, 3.8] | 0.01 |
| log UP:C at baseline | -1.0 [-1.6, -0.5] | <0.001 | -1.0 [-1.5, -0.4] | <0.001 |
| log2 uEGF/Cr at baseline | 6.2 [3.0, 9.3] | <0.001 | --- | --- |

Normalization was performed using median-interquartile range age-normalization of uEGF/Cr based on values from data from healthy children in a study from Meybosch, et al.(29, 30) Other variables tested for inclusion in the model are sex, ethnicity, kidney disease duration at baseline, and prior therapy at baseline.

Abbreviations: eGFR=estimated glomerular filtration rate (mL/min/1.73m^2^); EGF=epidermal growth factor; uEGF/Cr=urinary EGF: creatinine ratio (ng/mg); UP:C=urine protein: creatinine ratio (g/g)

Supplemental Table 2. Sensitivity analysis of age-normalized uEGF/Cr. Adjusted longitudinal linear mixed-effects models of eGFR over time. Results restricted to pediatric participants with kidney biopsy. (n=118 participants, 1,164 observations). Likelihood ratio test comparing Model 1 to Model 2: p <0.001

| Outcome = eGFR (mL/min/1.73m^2^) | **Model 1**  **(with uEGF/Cr)** | | **Model 2**  **(without uEGF/Cr)** | |
| --- | --- | --- | --- | --- |
|  | Estimate  [95% CI] | p-value | Estimate  [95% CI] | p-value |
| Main effects |  |  |  |  |
| Intercept | 37.7 | --- | 48.5 | *---* |
| Follow-up time (per year) | -2.1 [-5.4 to 1.2] | 0.22 | -1.7 [-3.1, -0.4] | 0.01 |
| Age (per year older) | -0.2 [-0.7 to 0.4] | 0.58 | -0.1 [-0.7, 0.5] | 0.74 |
| Race |  |  |  |  |
| Asian vs. White | 0.8 [-6.2 to 7.9] | 0.82 | -0.3 [-7.4, 6.9] | 0.94 |
| Black vs. White | 3.8 [-2.5 to 10.0] | 0.24 | 2.7 [-3.2, 8.5] | 0.37 |
| Native American vs. White | 7.3 [-5.3 to 19.8] | 0.26 | 9.9 [-3.3, 23.1] | 0.14 |
| Diagnosis |  |  |  |  |
| MCD vs. FSGS | 5.7 [2.3 to 9.0] | 0.001 | 8.7 [5.2, 12.1] | <0.001 |
| Winsorized eGFR at baseline | 0.6 [0.5 to 0.7] | <0.001 | 0.7 [0.6, 0.7] | <0.001 |
| log UP:C at baseline | 0.8 [-1.0 to 2.5] | 0.40 | 0.9 [-1.0, 2.8] | 0.33 |
| APOL1 (2 risk alleles vs 0/1) | -11.6 [-16.7 to -6.5] | <0.001 | -6.7 [-11.8, -1.5] | 0.01 |
| log Interstitial fibrosis (%) | -0.7 [-1.7 to 0.2] | 0.14 | -1.5 [-2.4, -0.5] | 0.003 |
| Log global sclerosis (%) | 0.3 [-0.9 to 1.5] | 0.65 | 0.5 [-0.7, 1.8] | 0.40 |
| log2 uEGF/Cr at baseline | 8.9 [-1.4 to 19.1] | 0.09 | --- | --- |
| Interaction with time (eGFR slope per year) (mL/min/1.73m2/year) |  |  |  |  |
| Age | 0.1 [-0.1 to 0.4] | 0.26 | 0.2 [-0.1, 0.4] | 0.15 |
| Race |  |  |  |  |
| Asian vs. White | -2.3 [-5.2 to 0.5] | 0.11 | -3.4 [-6.3, -0.4] | 0.03 |
| Black vs. White | 1.3 [-1.5 to 4.1] | 0.37 | -2.2 [-4.6, 0.1] | 0.07 |
| Native American vs. White | -8.4 [-13.2 to -3.7] | 0.001 | -10.1 [-15.1, -5.1] | <0.001 |
| log UP:C at baseline | -1.3 [-2.1 to -0.6] | <0.001 | -1.3 [-2.1, -0.5] | 0.001 |
| Log global sclerosis (%) | -1.1 [-1.6 to -0.6] | <0.001 | -0.8 [-1.3, -0.4] | 0.001 |
| log2 uEGF/Cr at baseline | 7.1 [2.8 to 11.5] | 0.001 | --- | --- |

Normalization was performed using median-interquartile range age-normalization of uEGF/Cr based on values from data from healthy children in a study from Meybosch, et al.(29, 30). Other variables tested for inclusion in the model are sex, race, ethnicity, diagnosis, kidney disease duration at baseline, prior therapy at baseline, interstitial fibrosis (IF) score, and EGF RNA expression.

Abbreviations: eGFR=estimated glomerular filtration rate (mL/min/1.73m^2^); EGF=epidermal growth factor; uEGF/Cr=urinary EGF: creatinine ratio (ng/mg); UP:C=urine protein: creatinine ratio (g/g)
